# Mismatch negativity (MMN) to pitch change is susceptible to order-dependent bias

**DOI:** 10.3389/fnins.2014.00180

**Published:** 2014-06-25

**Authors:** Juanita Todd, Andrew Heathcote, Lisa R. Whitson, Daniel Mullens, Alexander Provost, István Winkler

**Affiliations:** ^1^School of Psychology, University of NewcastleCallaghan, NSW, Australia; ^2^Priority Research Centre for Translational Neuroscience and Mental Health Research, University of NewcastleCallaghan, NSW, Australia; ^3^Schizophrenia Research InstituteDarlinghurst, NSW, Australia; ^4^Research Centre for Natural Sciences, MTA, Institute of Cognitive Neuroscience and PsychologyBudapest, Hungary; ^5^Institute of Psychology, University of SzegedSzeged, Hungary

**Keywords:** mismatch negativity (MMN), primacy, perceptual inference, auditory processing, stimulus specific adaptation (SSA)

## Abstract

Pattern learning facilitates prediction about upcoming events. Within the auditory system such predictions can be studied by examining effects on a component of the auditory-evoked potential known as mismatch negativity (MMN). MMN is elicited when sound does not conform to the characteristics inferred from statistical probabilities derived from the recent past. Stable patterning in sequences elevates confidence in automatically generated perceptual inferences about what sound should come next and when. MMN amplitude should be larger when sequence is highly stable compared to when it is more volatile. This expectation has been tested using a multi-timescale paradigm. In this study, two sounds of different duration alternate roles as a predictable repetitive “standard” and rare MMN-eliciting “deviation.” The paradigm consists of sound sequences that differ in the rate at which the roles of two tones alternate, varying from slowly changing (high stability) to rapidly alternating (low stability). Previous studies using this paradigm discovered a “primacy bias” affecting how stability in patterning impacts MMN amplitude. The primacy bias refers to the observation that the effect of longer-term stability within sequences only appears to impact MMN to the sound first encountered as deviant (the sound that is rare when the sequence commences). This study determines whether this order-driven bias generalizes to sequences that contain two tones differing in pitch. By manipulating (within-subjects) the order in which sounds are encountered as deviants the data demonstrate the two defining characteristics of primacy bias: (1) sequence stability only ever impacts MMN amplitude to the first-deviant sound; and (2) within higher stability sequences, MMN is significantly larger when a sound is the first compared to when it is the second deviant. The results are consistent with a general order-driven bias exerting modulating effects on MMN amplitude over a longer timescale.

## Introduction

Bayesian models of perception stipulate that learning is fundamentally linked to the ability to continuously update the brain's estimates of conditional probabilities (Mathys et al., 2011). The accuracy of these estimates determines the accuracy of predicting upcoming events. Therefore, Bayesian updating is considered especially important in the dynamic analysis of a sequence of sensory data, such as a train of sounds. The mismatch negativity (MMN) component of the auditory evoked potential provides a powerful tool with which to test Bayesian models (Näätänen et al., 1978; for a review, see Näätänen et al., 2011). MMN occurs when a sound violates some regular pattern within a sequence of sounds. Repetition of the pattern leads to the formation of what has been termed a “prediction model”- a memory containing information about sound characteristics and their transitions (Winkler et al., 1996a; Winkler, 2007). The learned pattern enables the brain to infer the most likely subsequent state of brain activation to follow the present state, in other words, to form predictions about what stimulus should come next based on a dynamically updated probabilistic inference. MMN is evoked when the prediction does not match the next state encountered.

Like most inferences and expectations, perceptual inferences underlying MMN are weighted by confidence in the prediction (Winkler, 2007; Winkler et al., 2009). MMN amplitude to prediction errors is largest when prediction confidence is high and lowest when the pattern has been found to be less reliable or stable. In Bayesian terms, confidence is referred to as precision and reflects stability in the pattern (Friston, 2005). Although prediction models form rapidly (within as few as 1–2 repetitions of a regularity, Cowan et al., 1993; Bendixen et al., 2007), precision estimates have to accumulate over longer time periods (Winkler et al., 1996b). Bayesian models have been highly successful in explaining experimental data on MMN and in generating hypotheses about the neural architecture supporting perceptual inferences (Garrido et al., 2009; Lieder et al., 2013). However, our previous research has discovered a powerful bias in how precision affects MMN (e.g., Todd et al., 2011, 2013a). In the current study, we tested the generality of this bias effect.

**Figure 1:**
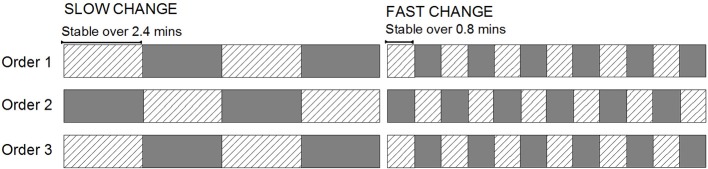
**A diagrammatic representation of the structure of conditions presented within the sound sequence**. The cross-hatched rectangles represent one deviant block type and the grayed rectangles, the other. For half the participants (Low-High-Low group), the grayed rectangles were “high-pitch deviant” blocks where the 1000 Hz tone was the standard and the 1500 Hz tone was the rare deviant and the hatched-pattern rectangles represent the reversed (“low-pitch deviant”) blocks. For the other half of the participants (High-Low-High group), the association between the rectangle fill and the standard/deviant configuration is the opposite.

In our previous studies, a two-tone multi-timescale sequence has been utilized to explore how the extraction of information about longer-term stability affects perceptual inferences about local patterns. The design was motivated by the aim of generating an index of the period of time over which past input would have an impact on confidence in current predictions (Todd et al., 2011). In the multi-time scale sequence two tones differing in their duration alternated roles as a highly repetitive “standard” (*p* = 0.875) and a rare “deviant” (*p* = 0.125). The key manipulation in this study was that the two tones alternated roles at different rates in separate sequences (see Figure 1, Order 1 for an example). In slower changing (more stable) sequences the roles changed every 2.4 min (organized into four blocks changing every 480 tones). This created a sequence with relatively high stability of the standard/deviant configuration. In contrast, the faster changing (more volatile) sequences featured roles alternating every 0.8 min (twelve blocks changing every 160 tones), producing a sequence with comparatively lower stability of the standard/deviant configuration. Due to the highly dynamic updating of transition statistics (see also Winkler et al., 1996a; Sussman and Winkler, 2001), MMN was always elicited when a tone that had been previously encountered as a frequent standard later appeared as a rare deviant.

The results of several studies employing duration as the deviating feature have revealed that the assumption that higher stability of the standard/deviant configuration leads to higher MMN amplitudes is only partially correct (Todd et al., 2011, 2013a,b; Mullens et al., 2014). MMN elicited to the sound first encountered as the devian*t* (that is, the sound that is rare when the sequence begins; hereafter called *first deviant*) is indeed larger in slow than faster changing sequences. This finding is consistent with higher confidence/precision in prediction models for the sequence with higher stability. However, MMN to the sound first encountered as standard when it is later encountered as a deviant (hereafter called the *second deviant*) was found to be of equal size in the slower and faster changing sequences. This observation was highly surprising given that the probability information about the two tones is in fact completely identical within the sequences. The only difference between them is which sound was the first encountered as common and which was first encountered as rare. This order-driven bias was present whether the short tone or the long tone was the first deviant showing that it was indeed linked to the first roles assigned to tones and not to the tone properties *per se*. Furthermore, the effect remained significant even when the history of exposure to sounds was balanced between the two sounds by removing the first and last blocks of the sequences to make sure that the effect was not a product of the responses to deviants obtained in the first block, only (Todd et al., 2011). Within a Bayesian framework, this difference in how MMN is affected by sequence stability appears to indicate that precision weightings affect inferences for only one of the two contexts (i.e., standard-deviant configurations)—the one first encountered. We term this effect, which appears as a tone by sequence interaction in the two-tone multi-timescale paradigm, a *primacy bias*. Primacy bias is clearly at odds with assumptions that the MMN system reflects a low-level filtering process that is slave to environmental statistics (Wacongne et al., 2012).

Primacy bias appears to indicate that precision only modulates MMN amplitude for the context encountered first. In a closer inspection of the phenomenon, Todd et al. (2013b) split the data from Todd et al. (2013a) into halves for each block of the slow and fast alternating sequences to explore how MMN amplitude changed from the beginning to end of blocks making up each sequence. Based on the assumption that confidence/precision affects the MMN amplitude, it should increase across the duration of a block as confidence builds up with the increased stability in the pattern. For the slow alternation of the standard/deviant roles (the first sequence encountered in the multi-timescale paradigm; see Figure 1), MMN to the *first deviant* was large in the first-half of the blocks and remained large over the second half. In sharp contrast, MMN to the *second deviant* was small right after the transition but grew significantly by the second half. In other words, initial confidence in the model of the first context (standard/deviant configuration) was much higher at transition points than confidence in the model of the second (reversed-roles) context. For the fast alternation of the standard/deviant roles (the second sequence encountered in the multi-timescale paradigm; see Figure 1), MMN was large over both halves for the *second deviant* but was small initially and increased by the second half for the *first deviant*. That is, the MMN amplitude pattern found for the slow alternation sequences reversed in the fast alternation sequences. This means that the differential sequence effect on MMN amplitude, which defines primacy bias (slow MMN > fast MMN for the first deviant and slow MMN = fast MMN for the second deviant) was driven by differences in MMN amplitude at transition points in the sequence when the roles of the sounds had recently switched (Todd et al., 2013b).

All previous studies examining the primacy bias have employed a temporal difference between tones to elicit MMN. The present study was designed to test whether primacy bias would also be present for MMN to a pure tone-frequency deviation. From a learning perspective the bias should certainly transfer to this context as the same differences in probability of tone roles apply. However, the generality of this finding to a frequency change is not a trivial question when considering how inferences are implemented in the auditory system. Precision estimates are thought to affect cortical responsiveness via the modulatory influence of top-down or “backward” connections (Friston, 2005; Lieder et al., 2013). Within the MMN system, these are known to include feedback connections from secondary to primary auditory cortex and from frontal to secondary auditory cortices. Responsiveness in neurons coding the predicted stimulus characteristics is dampened while that to different stimulus properties is sensitized. Models of inferential processes suggest that sensory cortices extract probabilistic information over short time periods only (e.g., a few hundred milliseconds) and require the reciprocal connections to increasingly more rostral brain areas to exact longer-timescale information (Garrido et al., 2008; Kiebel et al., 2008). Backward connections are, therefore, also likely to be essential in expressing primacy bias, since the modulation of MMN amplitude must include information about sequence differences that emerges over a time scale on the order of minutes. Because the assumed general modulation of MMN reflecting such long timescale information must compete against local influences governed by shorter timescale probabilities, susceptibility to bias could differ for spectral and temporal sound features, such as pitch vs. duration, just as the respective feature-specific MMNs differ from each other (see, e.g., Giard et al., 1995; Alain et al., 1999; Takegata et al., 2001).

Stimulus-specific adaptation (SSA) is a possible phenomenon underlying the differences between the encoding of regularities based on primary sound features. SSA is likely to indirectly contribute to the evoked potential measured as MMN (Ulanovsky et al., 2003; Winkler et al., 2009). Neurons exhibiting SSA radically reduce their response to a sound repeated a few times but they vigorously respond to sounds that differ from the repeated sound (hence the stimulus-specificity of the adaptation). SSA has been primarily observed for tones differing in frequency (pitch), and has been found at multiple levels in the auditory pathway including primary auditory cortex, medial geniculate body of the thalamus, and the inferior colliculus of the brainstem (for review Escera and Malmierca, 2014). Whether subcortical SSA can be observed for other stimulus features is less clear (Ayala and Malmierca, 2013) and the balance of evidence currently suggests that it is not present for more complex violations such as patterning emergent over a longer time course (i.e., high-high tonal deviants within a high-low alternating pattern, Cornella et al., 2012).

Encoding of sound duration is more complex than frequency in that there is no simple equivalent of the tonotopic mapping present for frequency at multiple levels of the auditory pathway. Further, deviance detection (as measured by MMN) is confined to the initial ca. 300 ms long segment of long unchanging sounds (Grimm and Schröger, 2005; Weise et al., 2010) suggesting that sound duration is derived differently for short vs. long and unchanging vs. structured sounds. Sound duration is processed in a distributed manner requiring different portions of the sound to be decomposed and recompiled across a network of neurons before converging on “duration tuned neurons” located primarily in secondary auditory cortex (He et al., 1997; He, 1998). Although some form of SSA has been observed for repeated stimulus duration in primary auditory cortex (Farley et al., 2010), it is possible, given the more complex processing involved, that duration is not represented at lower levels of the auditory pathway to the same extent as frequency. Thus differences in SSA, a putative contributor to the MMN ERP response, may affect the interaction between lower-level shorter-timescale and higher-level longer-timescale influences on the MMN response. For example, although SSA has been observed to occur on multiple timescales and over many seconds (Ulanovsky et al., 2003; Costa-Faidella et al., 2011), the multi-time scale protocol demonstrates changes in response to both standards and deviants that extend into many minutes (e.g., the halves analysis mentioned above demonstrated that suppressed response to repetitive standards continues to increment from 1.2 to 2.4 min, Todd et al., 2013a). These effects exceed the range known for SSA and are likely to reflect modifications in confidence/precision estimates that are embodied in top-down influences. If altered responsiveness to sounds for shorter timescales is stronger for frequency than duration due to the influence of multiple levels of SSA, it is entirely possible that primacy bias may have less or no influence over MMN to frequency deviations. Therefore, the present study uses the prototypical multi-timescale sequence but replaces duration deviance with deviation in frequency to determine the generality of the mechanisms underlying the primacy bias.

### Participants

A total of 30 participants (aged 18–34 years, mean = 23 years, *SD* = 2 years) were recruited. The participant group consisted of 6 males and 24 females, and all were recruited from the first year Undergraduate Psychology student body at the University of Newcastle and volunteers from the community. Participants were excluded if they were under 18 or over 35 years of age, were diagnosed or being treated for a mental illness, had a first degree relative with schizophrenia, regularly used recreational drugs, consumed alcohol regularly and heavily, had a history of neurological disorder, head injury or surgery, or a hearing impairment. Remuneration was offered as course credit to Psychology students and cash reimbursement ($20AUD) to community volunteers. Written informed consent was obtained from all participants.

### Stimuli and sequences

The protocol used replicates that in Todd et al. (2013a) with the exception of changes in the sound properties. Two sounds were organized into two different block types characterized by different sound probabilities. In low-pitch deviant blocks, 1500 Hz sounds were highly probable (*p* = 0.875) and 1000 Hz sounds were rare deviants (*p* = 0.125). In the high-pitch deviant blocks the probabilities were reversed (1000 Hz presented at *p* = 0.875 and 1500 Hz presented at *p* = 0.125). The sequences are depicted in Figure 1. Both sounds were pure tones 60 ms in duration with a 5-ms linear rise/fall time and they were presented binaurally over headphones at 75 dB SPL. In all sequences the sounds were presented at a regular 300 ms stimulus onset asynchrony.

Both low- and high-pitch deviant blocks were presented with slow and fast block-alternation speeds in separate sequences. The structure of sequences replicates that used previously (Todd et al., 2011, 2013a; Mullens et al., 2014). The slow sequence contained 1920 sounds in blocks that alternated every 480 tones, creating a block length or standard stability of 2.4 min (two repeats of each block). The fast sequence contained the same number of sounds, but the blocks alternated every 160 tones creating a block length or standard stability of 0.8 min (six repeats of each block). Each sequence lasted 9.6 min in total.

The sequences were presented in three pairs (“orders”) with fast following slow alternating versions in each case. This design facilitates an examination of whether the primacy bias pattern reverses with tone-order. In 15 participants (the Low-High-Low group), the sequence was presented with order 1 and 3 sequences beginning with the low-pitch deviant blocks and order 2 with the high-pitch deviant block. In the remaining 15 (the High-Low-High group), the role of the high and low tones in the orders were reversed. Order was balanced across participants alternating with recruitment order (3 males in each subgroup and mean age of 23 in both subgroups). A 5 min break was enforced between orders and shorter 1–2 min breaks occurred between the two sequences within each order (total testing time approximately 1 h and 15 min).

### Procedure

All participants completed a screening interview to determine that all inclusion criteria were met. An audiometric screening using a pure tone audiometer (Earscan ES3S Manual Screening Auditometer) across 500–4000 Hz was used to check for adequate hearing (thresholds = 25 dB SPL) and exclude to hearing loss. Participants were then fitted with a Neuroscan Quickcap with tin electrodes. The continuous EEG was recorded on a Synamps 2 Neuroscan system at 1000 Hz sampling rate (highpass 0.1 Hz, lowpass 70 Hz, notch filter 50 Hz and a fixed gain of 2010). EEG data were recorded from 10 electrode locations (FZ, FCZ, CZ, PZ, F3, FC3, C3, F4, FC4, C4 in accordance with the 10–20 system plus left mastoid, right mastoid) referenced to the nose. Vertical and horizontal electro-oculograms were monitored by electrodes above and below the left eye, and 1 cm lateral from the outer canthi of each eye to monitor blinks and eye movements. Impedances were reduced to below 5 kΩ before recording commenced. Sequences were presented over headphones (Sennheiser HD280 pro) while the participant viewed a silent DVD presentation with subtitles and was asked to remain as still as possible (to minimize movement artifact in the ERP) and to ignore the sounds and focus attention on the movie.

### Data analysis

The continuous EEG recording was first examined offline for major artifacts and corrected for eye blinks using the procedures in Neuroscan Edit Software. This method applies a regression analysis in combination with artifact averaging (Semlitsch et al., 1986). The average artifact response algorithm was assessed for adequacy (more than 30 sweeps in the average and < 5% variance) and applied to the continuous data file. Each file was epoched from 50 ms pre-stimulus to 300 ms post-stimulus.

Epochs were baseline corrected to the pre-stimulus interval and then averaged according to stimulus type. Epochs containing voltage variations exceeding ±70 μV were excluded. Standard and deviant ERPs were averaged separately for the period equating to the first half of blocks (0–1.2 min for slow blocks and 0–0.4 min for fast blocks) and for second half of blocks (1.2–2.4 min for slow blocks and 0.4–0.8 min for fast blocks). All standard and deviant ERPs were digitally filtered with a lowpass of 30 Hz (best for examining exogenous components like N100; Kujala et al., 2007). MMN was computed by subtracting the averaged response to each standard from the averaged response to the corresponding deviant, separately for each sequence and block half. For example, the difference waveform for a 1500 Hz deviant for the first half data in fast change blocks was created by subtracting the ERP to the 1500 Hz standard in the first half of fast change blocks from the ERP to the 1500 Hz deviant tone in the first half of fast change blocks. This approach minimizes the contribution of physical differences between the standard and deviant sounds on the estimation of the MMN amplitude. The difference waveforms estimating the MMN was then filtered with a low pass of 20 Hz (lower cut-off recommended for MMN; Kujala et al., 2007). All MMNs were created with a minimum of 45 sweeps for the contributing deviant ERP. The actual minimum sweep count for any average in the Low-High-Low group deviants was 51 with mean sweeps for all deviant ERPs in excess of 58. The actual minimum sweep count for any average in the High-Low-High group deviants was 45 with mean sweeps for all deviants in excess of 56. There was no significant difference in the number of sweeps contributing to averages between either conditions or groups.

All ERPs were re-referenced to the averaged activity at the left and right mastoid sites to maximize signal to noise ratio (Joutsiniemi et al., 1998). The peak amplitude at Fz for each MMN produced during the first half of blocks was extracted from 50 to 200 ms post-stimulus. Peak amplitudes were measured rather than mean amplitudes of intervals, because the MMN latency varied across conditions. Peak latencies were, however, not analyzed in the current data, because the latencies are co-determined by genuine MMN latency effects as well as the ratio between the MMN and N1 amplitude. As the current parameters were not optimized to disentangle these effects, any interpretation of the pattern of peak latencies would lead to speculation. Future studies will address possible order-driven MMN latency effects. The choice to analyse MMN from first halves of each block was based on the results of Todd et al. (2013b) where the bias was only present at sequence transition points. In the present data set the interactions reported from analyses below were similarly not present in the MMNs acquired from the second half of sequence blocks and therefore analyses of first half data only is reported in the results section. Evidence of primacy bias was sought in a mixed model repeated measures ANOVA with a between-subjects factor of Group (High-Low-High, Low-High-Low) and within-subjects factors of Order (1, 2, 3), Sequence (fast, slow) and Tone (high, low). Subsequent analyses were used to test a-priori hypotheses that the primacy bias seen for MMN to duration deviant sounds (Todd et al., 2011, 2013a) would be replicated for frequency deviant sounds. The Greenhouse Geisser correction factor (ε) is reported where appropriate, together with the effect size (η^2^).

## Results

### Bias pattern 1: slow sequence MMN larger than fast sequence MMN for the first deviant only

**Figure 2:**
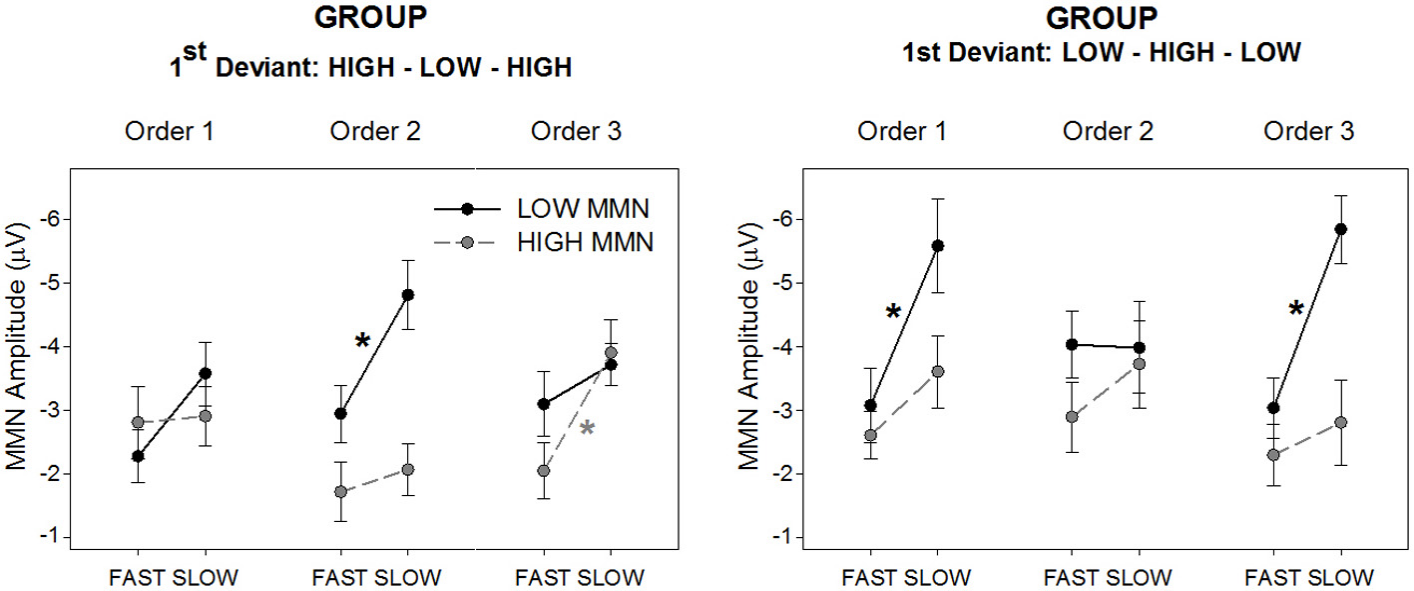
**Mean MMN amplitude for deviant high (gray/broken lines; “HIGH MMN”) and low (black full lines; “LOW MMN”) pitched tones, separately for the group hearing sequences with the High-Low-High 1st deviant order (left panel) and Low-High-Low 1st deviant order (right panel)**. The figure illustrates the MMN amplitude changes from the slow to the fast alternating sequences, separately for the three orders (*x*-axes). Asterisks represent significant fast vs. slow sequence difference of the mean MMN amplitudes yielded by paired *t*-tests (*p* < 0.05, at least). Error bars represent standard error of the mean.

**Figure 3:**
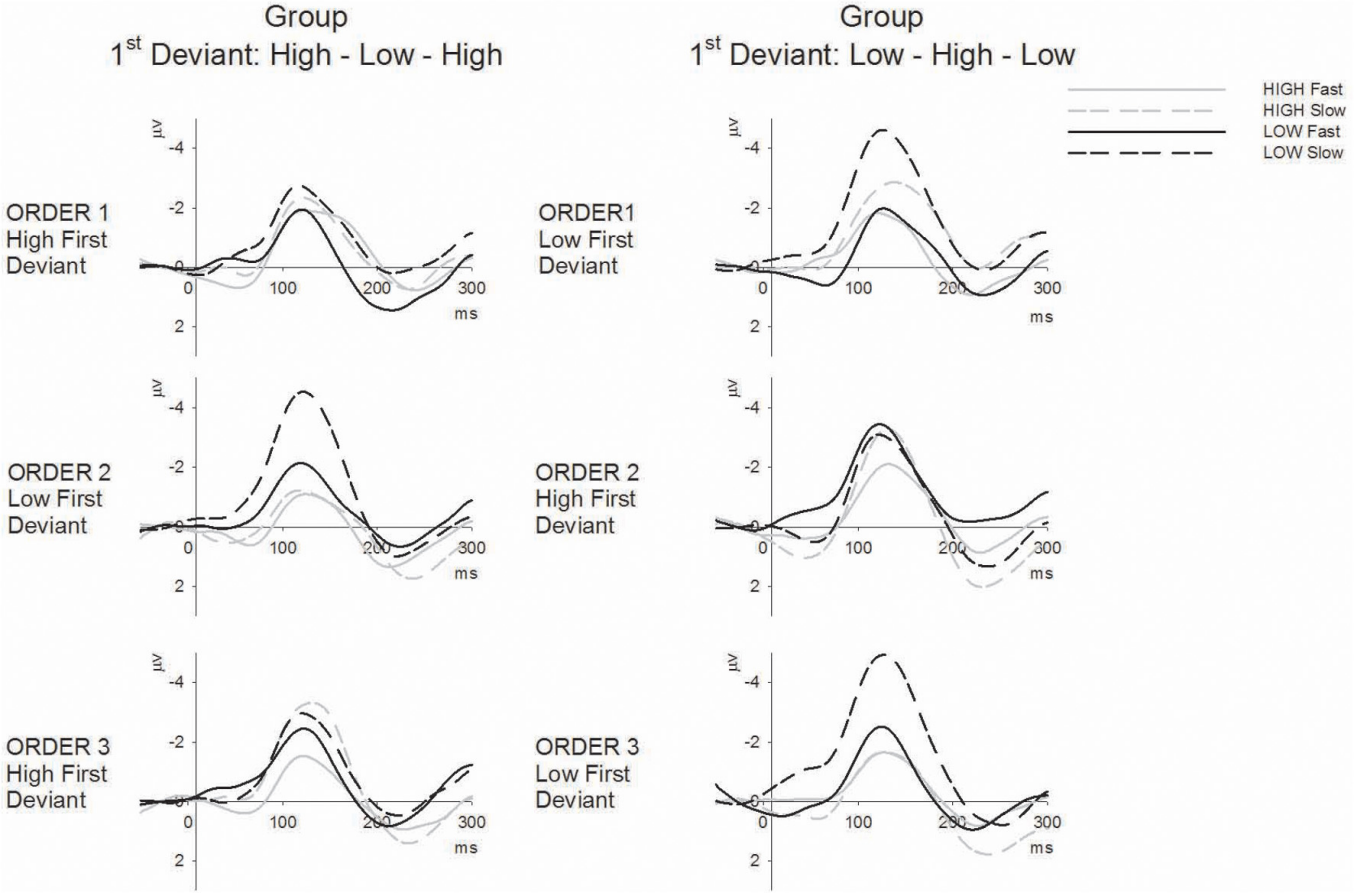
**ERP difference waveforms (deviant minus standard) for high tones (gray lines) and low tones (black lines) in fast (solid) and slow (broken) sequences separately for the High-Low-High 1st deviant order (left) and Low-High-Low 1st deviant order (right) groups across the three tone orders (columns)**.

The main hypothesis based on prior data for duration MMN was that there should be a sequence (fast vs. slow) by tone (high vs. low) interaction that is dependent on order (1, 2, 3). Since order was dependent upon group allocation, it was expected that this three-way interaction would be further modified by group. The omnibus ANOVA confirmed this four-way interaction between group, order, sequence, and tone [*F*_(2, 56)_ = 5.93, *p* < 0.01, ε = 0.87, η^2^ = 0.18]. Separate plots for each group are presented in Figure 2 showing the group mean amplitudes for each MMN to each tone across sequence type and order. The corresponding ERP difference waveforms for each group to each tone across sequence type and order are presented in Figure 3.

In previous studies the tone by sequence interaction characterizing the bias presented as larger MMN in slow than fast sequences for the first deviant only. A-priori paired *t*-tests confirm that significantly larger MMN in the slow than the fast sequence is only present for the tone first encountered as deviant within the given order (see Figure 2 asterisks and the visible differences in Figure 3). However the tone by sequence interaction (indicating that sequence effects differ significantly for the two tones) only reaches significance in order 2 for the High-Low-High group [Figure 2 left, *F*_(1, 14)_ = 5.23, *p* < 0.05, η^2^ = 0.27] and order 3 for the Low-High-Low group [Figure 2 right, *F*_(1, 14)_ = 7.09, *p* < 0.05, η^2^ = 0.34]; further, it is marginal in order 1 for the High-Low-High group [*F*_(1, 14)_ = 3.74, *p* < 0.05, η^2^ = 0.21]. In order 1 the Low-High-Low group produces main effects of tone and sequence [*F*_(1, 14)_ = 13.06, *p* < 0.005, η^2^ = 0.48 and *F*_(1, 14)_ = 33.07, *p* < 0.001, η^2^ = 0.70, respectively] but no interaction between them, and in order 2 this group shows no main effects or interactions. Finally, in order 3 the High-Low-High group produces a main effect of sequence [*F*_(1, 14)_ = 25.01, *p* < 0.001, η^2^ = 0.64] with a trend for MMN to both tones being larger in slow sequences.

### Bias pattern 2: pronounced order effects for slow sequence MMN

**Figure 4:**
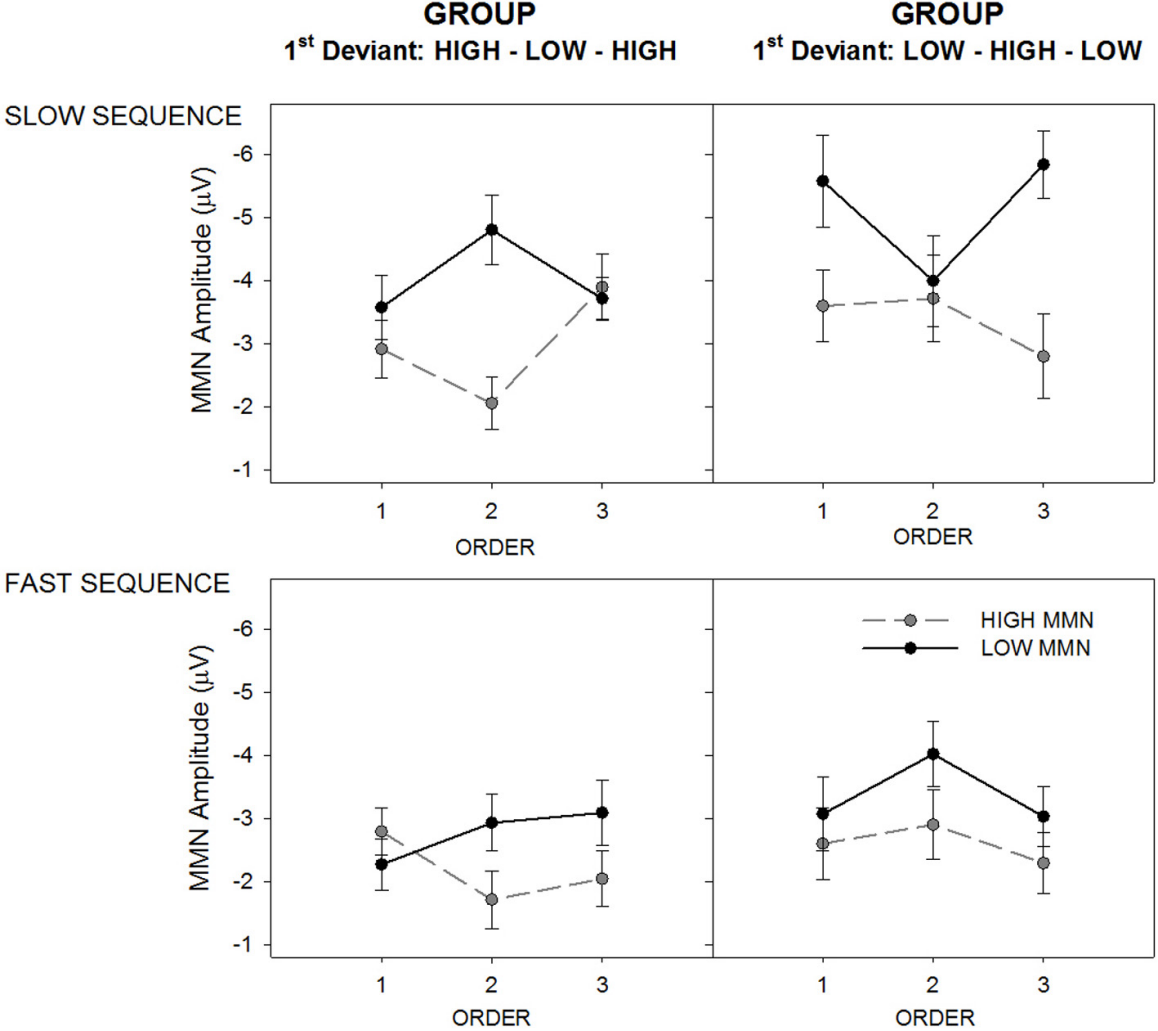
**Mean MMN amplitude for deviant high (gray/broken lines; “HIGH MMN”) and low (black full lines; “LOW MMN”) pitched tones, separately for the group hearing sequences with the High-Low-High 1st deviant order (left panels) and Low-High-Low 1st deviant order (right panels)**. The figure illustrates the MMN amplitude change across orders (*x*-axes), separately for the slow (top panels) and fast alternating sequences (bottom panels). Error bars represent standard error of the mean.

In prior studies order effects on MMN amplitude have generally been more pronounced in slow sequence data. A mixed model ANOVA was used to assess whether MMN in slow sequence data would be affected by whether a sound was encountered as the first or second deviant. The analysis revealed a three-way interaction between group, order, and tone for slow sequence data [*F*_(2, 27)_ = 9.53, *p* < 0.001, ε = 0.89, η^2^ = 0.47]. Figure 4 illustrates the interaction between order and sequence separately for the two tones and two groups. The three-way interaction was characterized by a significant quadratic trend clearly evident in Figure 4 [*F*_(1, 28)_ = 24.45, *p* < 0.001, η^2^ = 0.47] where MMN to a given tone type is always maximal when it was encountered as the first deviant. The manipulation of tone order between groups therefore results in opposing quadratic trends for the tone by order interactions.

The tone by order interaction was significant for the High-Low-High group [*F*_(2, 13)_ = 6.91, *p* < 0.05, ε = 0.99, η^2^ = 0.51] and marginal for the Low-High-Low group [*F*_(2, 13)_ = 3.47, *p* = 0.058, ε = 0.79, η^2^ = 0.20). In the High-Low-High group (Figure 3, left panel) there was a significant quadratic effect on MMN amplitude to high tones [*F*_(1, 14)_ = 5.37, *p* < 0.05, η^2^ = 0.27] and a marginal quadratic effect in the opposite direction on the low-tone MMN amplitude [*F*_(1, 14)_ = 4.48, *p* = 0.053, η^2^ = 0.24]. In the Low-High-Low group (Figure 3, right panel) there was a significant quadratic effect on MMN amplitude to low tones [*F*_(1, 14)_ = 10.89, *p* < 0.005, η^2^ = 0.44] but not on the high-tone MMN amplitude.

Fast sequence data produced a main effect of tone [*F*_(1, 28)_ = 14.44, *p* < 0.001, ε = 0.89, η^2^ = 0.34] modified by a marginal interaction with order [*F*_(2, 28)_ = 3.19, *p* = 0.051, ε = 0.96, η^2^ = 0.10]. In the bottom panel of Figure 4 it is clear that MMN to the low tone was generally larger than that to the high tone across orders and groups, but this was most apparent in order 2. The data therefore demonstrate that within the slow but not the fast sequences, MMN to a given tone will tend to be larger if it is first encountered as deviant than when it was encountered first as standard and later became a deviant (second deviant). Furthermore, this effect appears to be most pronounced for the sound encountered as deviant the very first time (i.e., for the low tone in the Low-High-Low group and for the high tone in the High-Low-High group).

## Discussion

The multi-timescale sequence protocol involves two tones alternating roles as a repetitive standard and a rare deviant with the alternation occurring at different rates in separate sequences. Models of perceptual inference predict that MMN should be elicited to all sounds when contextually encountered as rare deviations from a pattern but that the MMN amplitude should be larger in sequences where roles alternate slowly as opposed to rapidly due to volatility in the latter leading to lower precision or confidence in predictions (Friston, 2005; Winkler, 2007). The paradigm used here confirmed that these sequence effects on MMN are modulated by an order-dependent bias and that MMN elicited to a change in tone pitch is indeed affected by the same profound order-dependent bias as that has been previously observed for MMN to a deviance in sound duration.

The comparison of MMN in slow and fast changing sequences showed that MMN amplitude was only ever larger in the slower relative to faster changing sequence for the sound that was the *first deviant*. MMN size to the second deviant (i.e., the sound that was initially encountered as a repetitive standard then later became the deviant when roles reversed) did not differ significantly between the slow and faster changing sequences. This order-dependent effect on MMN amplitude is one of the key findings in all studies on the primacy bias (Todd et al., 2011, 2013a,b; Mullens et al., 2014). The results also revealed a second related pattern of order-driven bias when comparing the MMN amplitude change in slow sequences between the first vs. the second deviant. In both the High-Low-High and the Low-High-Low group, MMN amplitude was clearly affected by whether it was the first or second deviant in the slow- as opposed to a much lesser extent in the fast-alternation sequences (see Figure 4). In the slow-alternation sequences, MMN amplitude was always larger when the tone was the *first deviant* compared to when the same tone was the *second deviant*. Therefore, the order in which sounds were presented in standard and deviant roles had a profound impact on how sequence stability affected MMN size.

In summary, the present study replicated two elements of bias affecting MMN: (1) MMN to the sound first encountered as a deviant is more susceptible to modulation by sequence stability (i.e., the fast vs. slow sequence effects) than MMN to the sound that is first encountered as standard, and (2) within a slowly changing sequence, MMN to the first deviant is larger than to the second deviant. Both of these observations provide clear evidence that there are factors influencing MMN size beyond those accounted for in existing models of the underlying processes (Näätänen, 1984, 1990, 1992; Javitt et al., 1996; Schröger, 1997; May et al., 1999; Winkler, 2007; Garrido et al., 2009; Winkler et al., 2009; May and Tiitinen, 2010; Garagnani and Pulvermüller, 2011; Näätänen et al., 2011; Wacongne et al., 2012; Kaya and Elhilali, 2013; Schröger et al., 2013). In Introduction, we hypothesized that differences between processing tone frequency and duration (specifically SSA) may result in differences in the order-driven bias effect for these two features. The finding of similar effects suggests that the differences in processing these features do not result in substantial difference in the strength of the local contribution to MMN or that the top-down modulation is sufficiently strong, thus equalizing the possible local differences. The first alternative is compatible with the notion that the SSA contribution to MMN is indirect (Winkler et al., 2009) and therefore, differences found between SSA for the two features do not directly translate into differences in the local contribution to MMN.

In the present data set MMN in faster changing sequences was not affected significantly by sequence order but instead tended to be larger for the low pitched deviant independent of order. Although it is possible that this finding reflects a point of difference between duration and pitch change MMN, a failure to observe significant order biases in the faster changing sequences has been observed previously in duration MMN data (Mullens et al., 2014) and it was attributed to excessive volatility concealing any order-driven bias. The expression of bias is likely to reflect the impact of top-down modulation of local stimulus responsiveness. Under conditions of high volatility the influence of top-down effects is reduced (Friston, 2005; Lieder et al., 2013). In some datasets (e.g., Todd et al., 2013b), the influence of bias can indeed be observed on the MMN amplitude for fast changing sequences, but in others it is apparently not detectable (e.g., Mullens et al., 2014). Across studies it is becoming clear that order-driven effects are most reliably observed in slower changing sequences, which is consistent with the order-driven effects being expressed in sequences that allow a stronger top-down influence. The finding that primacy bias generalizes to pitch MMN provides evidence that this is truly a learning phenomenon that is anchored to the initial probabilities of the two sounds in the unattended task-irrelevant multi-timescale sequences. A recent study reveals that order-dependent biases are also evident when the sounds are attended: The N2b component for the auditory ERP elicited to rare target stimuli is significantly reduced if the sequence of sound contains a higher initial concentration of targets compared to when targets are evenly spread over time (Kotchoubey, 2014). In Kotchoubey's (2014) “primacy sequence” the targets (*p* = 0.22 overall) were initially encountered as the more probable sound while in a “classic sequence” the targets were evenly spread (*p* = 0.22 overall). The early concentration of targets meant that targets were in fact rarer later in the primacy sequence than in the classic sequence, which should have enhanced the N2b to later targets in the primacy relative to the classic sequence (Squires et al., 1976), and yet the reverse pattern was observed. These observations combined with the results of the present study indicate that the initial probabilities with which we encounter sound, whether attended and task-relevant (as in Kotchoubey, 2014) or ignored and task-irrelevant (as in the present study), profoundly distort the way the brain responds to them for a prolonged period of time. The influence of factors thought to have a key impact on brain responses, predictive confidence or precision in the case of MMN and rarity of targets in the case of N2b (Banquet et al., 1981), appear to be overpowered by these order-driven/frequency effects.

In conclusion, we have shown that the primacy bias, which has been previously observed to affect tones of two durations that alternate roles as a common standard and rare deviant, is also apparent for sequences comprising two tones separated in pitch. The findings of the present study support the contention that primacy bias is a phenomenon affecting auditory perceptual inferences generally—i.e., they are based on a general learning process. The reasons why the bias occurs, and which brain regions participate in generating the bias, remains an open question. However, it is becoming increasingly clear that order driven phenomena can have a profound impact on even the lowest levels of auditory relevance filtering and the potential impact should be considered in experimental designs.

### Conflict of interest statement

The authors declare that the research was conducted in the absence of any commercial or financial relationships that could be construed as a potential conflict of interest.
